# Case report: Acute hepatitis in neonates with COVID-19 during the Omicron SARS-CoV-2 variant wave: a report of four cases

**DOI:** 10.3389/fped.2023.1179402

**Published:** 2023-05-05

**Authors:** Jing Wang, Wei Hu, Kexin Wang, Rong Yu, Liwen Chang, Zhihui Rong

**Affiliations:** Department of Pediatrics, Tongji Hospital, Tongji Medical College, Huazhong University of Science and Technology, Wuhan, China

**Keywords:** neonate, SARS-CoV-2, Omicron variant, COVID-19, acute hepatitis, liver injury, transaminase

## Abstract

**Background:**

Severe Acute Respiratory Syndrome Coronavirus 2 (SARS-CoV-2), first emerging in December 2019 and continuously evolving, poses a considerable challenge worldwide. It was reported in the literature that neonates had mild upper respiratory symptoms and a better outcome after Omicron SARS-CoV-2 variant infection, but there was insufficient data about complications and prognosis.

**Case Presentation:**

In this paper, we present the clinical and laboratory characteristics of four COVID-19 neonate patients with acute hepatitis during the Omicron SARS-CoV-2 variant wave. All patients had a clear history of Omicron exposure and were infected via contact with confirmed caregivers. Low to moderate fever and respiratory symptoms were the primary clinical manifestations, and all patients had a normal liver function at the initial stage of the course. Then, the fever lasted 2 to 4 days, and it was noted that hepatic dysfunction might have occurred 5 to 8 days after the first onset of fever, mainly characterized by moderate ALT and AST elevation (>3 to 10-fold of upper limit). There were no abnormalities in bilirubin levels, blood ammonia, protein synthesis, lipid metabolism, and coagulation. All the patients received hepatoprotective therapy, and transaminase levels gradually decreased to the normal range after 2 to 3 weeks without other complications.

**Conclusions:**

This is the first case series about moderate to severe hepatitis in COVID-19 neonatal patients via horizontal transmission. Besides fever and respiratory symptoms, the clinical doctor should pay much attention to evaluating the risk of liver function injury after SARS-CoV-2 variants infection, which is usually asymptomatic and has a delayed onset.

## Introduction

Severe Acute Respiratory Syndrome Coronavirus 2 (SARS-CoV-2), first emerging in December 2019 and continuously evolving to different variants through gene mutations, brought tremendous challenges and burdens to global public health (1–3). Since a new Omicron variant was discovered in November of 2021 in South Africa, it quickly spread worldwide and has replaced the Delta variant as the dominant pandemic strain (4, 5). The Omicron variant has identified a much greater number of mutations than any previous strains. The mutations, mainly on the spike protein of the virus, significantly increase binding affinity to human ACE2 receptors, contributing to immune evasion property and transmission fitness (6, 7). Compared with previous strains, the Omicron variant is more transmissible and easy to infect younger individuals (8). The literature on neonates with Omicron variant infection is limited; most of them are perinatal cases (9, 10). Xu et al. reported mild upper respiratory symptoms and a better outcome for neonates during the Omicron variant wave in Shanghai (11). However, there is insufficient data on the complication and prognosis for neonates with Omicron variant infection. As far as we know, there have been few reports about liver injury accompanied by Omicron variant infection in neonates, which is a rare symptom. Therefore, we present the clinical characteristics and outcomes of four Omicron-infected neonate patients with acute hepatitis in this study.

## Case presentation

### Case 1

A 27-day-old boy presented to the emergency room with a 7-hour history of fever. After giving superficial skin cooling at home, the baby remained febrile. The peak temperature was 38.5°C. The baby had a poor appetite and symptoms of sneezing and a stuffy nose without vomiting and coughing. He was a term baby without extraordinary perinatal history. His parents had a history of sore throat and cough for several days during the Omicron epidemic, but they didn't test for SARS-CoV-2. After admission to our NICU, he was febrile at 38.3°C with a respiratory rate of 52 breaths/min and oxygen saturation of 95%–99% while breathing ambient air. His examination was remarkable for congested nares, clear rhinorrhea, and mild subcostal retractions. There were coarse breath sounds in all lung fields. No murmur of the heart could be heard. The liver was palpated at 1 cm–2 cm under the costal margin (consistent with age), and the spleen was not palpated. Laboratory data revealed that the total WBC count and the proportions of the major leukocyte subsets in peripheral blood were normal. Blood gas, C reactive protein (CRP), and transaminase levels were in the normal range. RNA test for SARS-CoV-2 from a throat swab was positive. Then, the baby was given physical cooling and nasal secretion removal to keep the airway clear (see Table 1).

**Figure 1:**
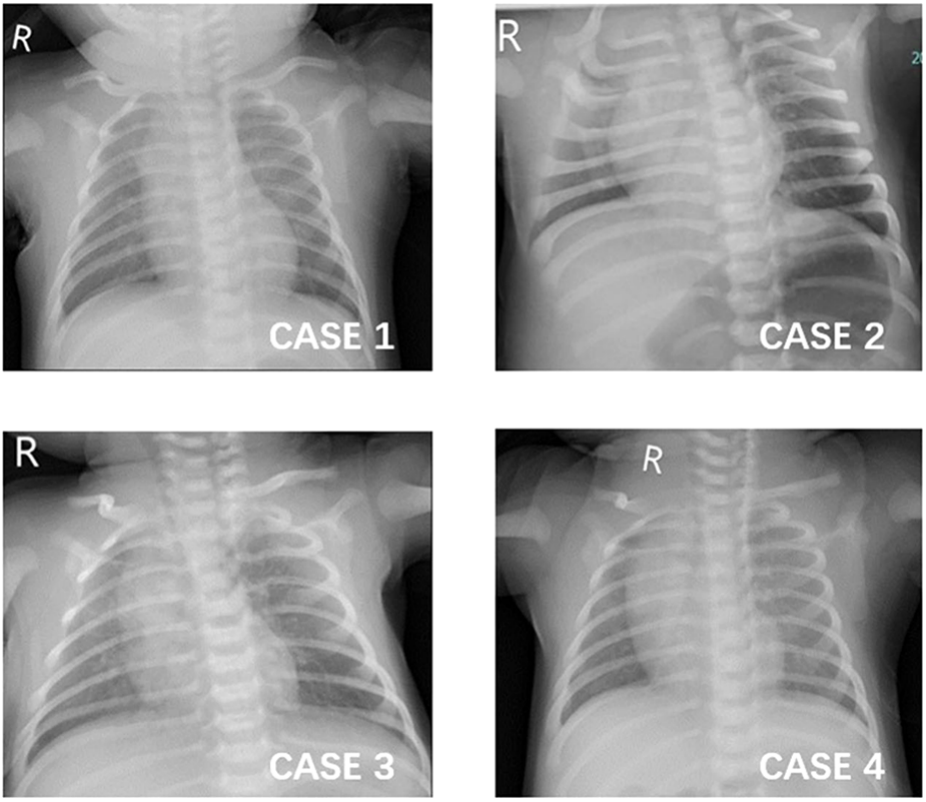
Chest x-ray images of the four patients. (1) CASE 1: increased and blurred bilateral lung markings. (2) CASE 2: increased and blurred bilateral lung markings. (3) CASE 3: increased and blurred bilateral lung markings and patchy shadows on the right upper lung field. (4) CASE 4: increased and blurred bilateral lung markings.

The baby presented febrile once daily in the following four days, and the peak temperature decreased from 38.4 to 38°C. Meanwhile, he developed a cough and sputum, along with a dropping of SpO2 to 80% when feeding. His lung demonstrated scattered crackles (see Figure 1). Because of continuous fever and pneumonia, more investigations were performed. The total WBC count and the proportions of the major leukocyte subsets were still in the normal range. CRP was 4.3 mg/L (0–10 mg/L). Anemia was noticed: RBC was 2.73 × 1,012/L, hemoglobin was 91.0 g/L, and hematocrit was 27.4%. Alanine transaminase (ALT) was 88 U/L(≤41 U/L), aspartate transaminase (AST) was 180 IU/L(≤40 U/L), and glutamyltranspeptidase (γ-GT) was 109 U/L(6–42 U/L). Further, pathogens tests identified that parainfluenza virus RNA was positive, but there was no evidence of infection for other pathogens such as RSV, EBV, TORCH, ECHO virus, Coxsackie virus (CA16/CVB), influenza A/B/H1N1/H3N2, adenovirus, mycoplasma, chlamydia, HIV, Human metapneumovirus, rhinovirus, and hepatitis B/C virus. Ultrasonography for the heart and abdomen was normal. Therefore, the baby was given the nebulization treatment of budesonide and ipratropium bromide solution and given Glutathione for hepatoprotection. In addition, he inhaled oxygen intermittently to avoid hypoxia when feeding.

On the 7th day after admission, the patient was no longer febrile but was still coughing and had nasal congestion. On the 10th day, respiratory symptoms improved greatly, and we re-tested the liver function after 5 days of hepatoprotective treatment. The transaminase level elevated markedly. ALT was up to 645 U/L(≤41 U/L), AST increased to 480 IU/L(≤40 U/L), and γ-GT was 491 U/L(6–42 U/L). However, bilirubin, blood ammonia, blood glucose, lipids, lactate, coagulation function, and albumin levels were within the normal range. The patient's perinatal medical history and family history were tracked carefully, and the possibility of inherited metabolic liver disease was ruled out. Furthermore, the patient's history of drug exposure before/after admission was also reviewed, and there was no evidence of drug-induced liver injury. So, virus infection may contribute to liver injury and the elevation of transaminase levels. When the immune system clears the virus, the injury should be alleviated. Thus, we only administered glycyrrhizin and bicyclol to promote recovery.

**Figure 2:**
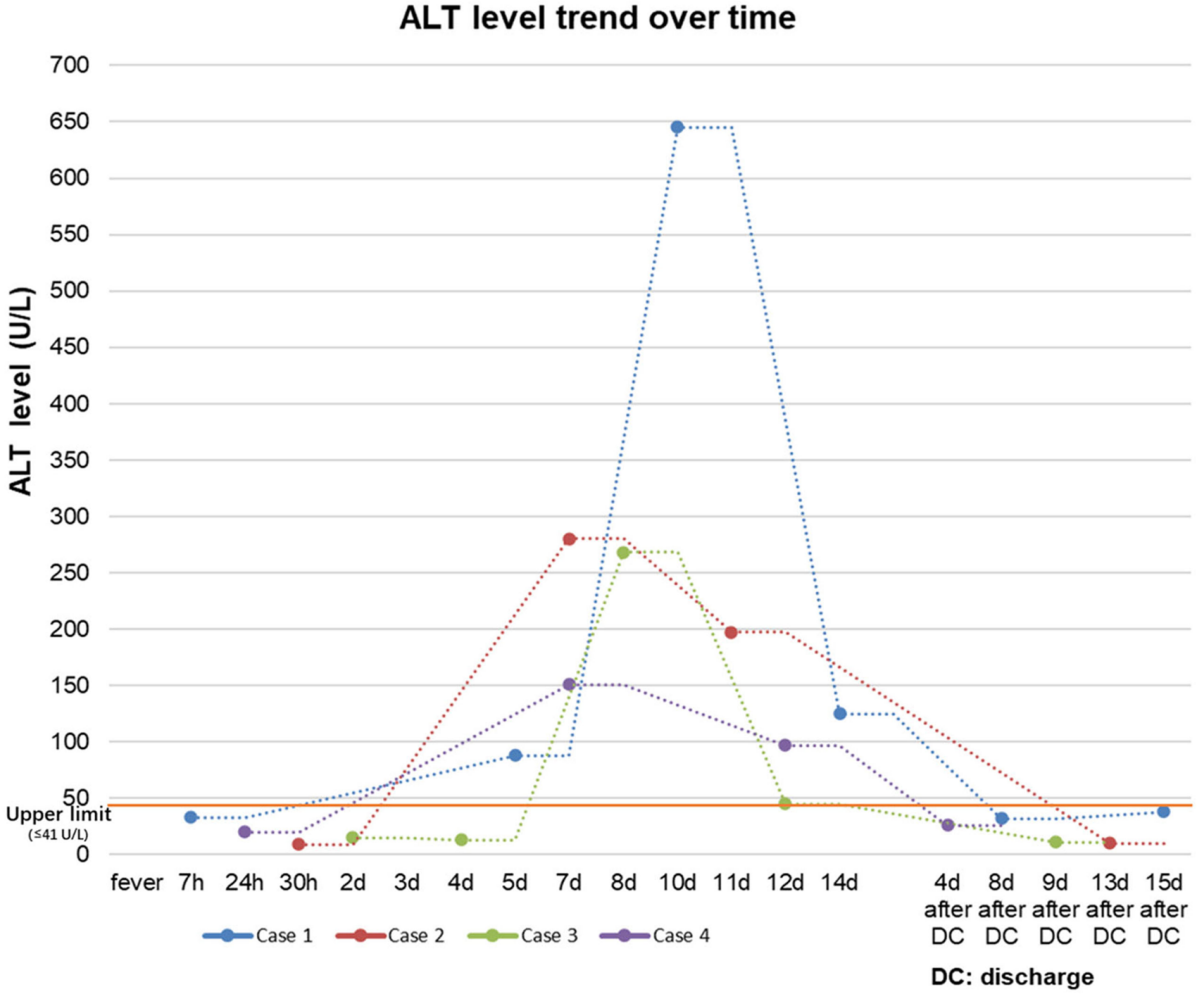
ALT level trend over time of the four patients. All patients had normal liver function at the initial stage of the course. ALT elevation (>41 U/L) occurred 5 to 8 days after the first onset of fever, then reached 3 to 10 folds of the upper range. All the patients received hepatoprotective therapy, and transaminase levels gradually decreased to the normal range after 2 to 3 weeks.

On the 14th day, the baby looked well with the normal physical examination. Laboratory tests demonstrated that ALT decreased to 125 U/L(≤41 U/L), AST was 44 IU/L(≤40 U/L), and γ-GT was 283 U/L(6–42 U/L). He continued to take glycyrrhizin and bicyclol after being discharged home. The liver transaminase level returned to normal on 8 and 15 days after discharge (see Table 2 and Figure 2).

**Table 1:** Summary of clinical presentation and outcome of COVID-19 neonate cases with acute hepatitis.

No.	Age of onset (d)	GA (g)	BW (g)	Wt (g)	Epidemiological exposure history			Comorbidity	Symptom	Chest radiology	Ultrasound	Peak Temp (°C)	Fever duration (days)	Special abnormal finding	Pathogens		Treatment	Recovery/Outcome	
					Epidemic	Contact history	Mother's vaccination								SARS-CoV-2	Others*		Respiratory symptoms(d)	Liver function**
1	27	38 weeks	3,400	4,640	Omicron variant	The parent had a history of sore throat and cough for several days without COVID-19 test.	no	No	Fever; Anorexia; stuffy nose; sneeze; cough and sputum; SpO2 dropped to 80% when feeding	x-rays: increased and blurred bilateral lung markings	Cardiac/abdominal US (−)	38.5	7 days	Found elevated liver enzymes after 5 days of fever;	PCR for RNA (+)	Parainfluenza virus (oropharyngeal swab)	Superficial skin cooling; oxygen inhalation when feeding; nebulization; hepatoprotective drugs	10 days	21 days
2	7	40w^+6^	4,020	3,600	Omicron variant	Mother confirmed infection	Two does	Congenital Hydronephrosis (bilateral, mild); right renal calculus (3.1 × 2.7 mm)	Fever; choking on milk, spitting; stuffy nose; cough occasionally	x-rays: increased and blurred bilateral lung markings	Cardiac US: PFO 2.6 mm (left-to-right shunt); EF: 68%; kidney US: mild bilateral hydronephrosis and calculus in the right kidney(3.1 mm × 2.7 mm); liver/gallbladder/spleen US: (−)	38.7	3 days	Found elevated liver enzymes after 7 days of fever	RNA and antigen: (+)	None	Superficial skin cooling; piperacillin/tazobactam; hepatoprotective drugs	9 days	18 days
3	15	40 w	3,350	3,530	Omicron variant	Caregivers confirmed infection	no	No	Fever; stuffy nose and runny nose; mild cough; coarse and wet rales	x-rays: increased and blurred bilateral lung markings, and patchy shadows on the right upper lung field.	Cardiac/abdominal US (−)	38.3	3 days	Found elevated liver enzymes after 8 days of fever	(+)	None	Superficial skin cooling; piperacillin/tazobactam; hepatoprotective drugs	11 days	14 days
4	24	36w^+4^	3,050	4,680	Omicron variant	Mother confirmed infection	no	No	Fever, anorexia, cough and sputum; watery diarrhea; cough occasionally; skin markings on lower extremity	x-rays: increased and blurred bilateral lung markings	Cardiac/abdominal US (−)	38	3 days	Found elevated liver enzymes after 7 days of fever	(+)	None	Superficial skin cooling; empirical antibiotics for 36 h; hepatoprotective drugs;	10 days	10 days

* Others: RSV, EBV, TORCH, ECHO virus, Coxsackie virus(CA16/CVB), influenza A/B/H1N1/H3N2, adenovirus, mycoplasma, chlamydia, HIV, Human metapneumovirus, rhinovirus, and hepatitis B/C virus.

** Liver function: the duration of ALT decreasing to the normal range.

**Table 2:** Laboratory data on presentation of COVID-19 neonate cases with acute hepatitis.

No.		Duration after fever	ALT (≤41 U/L)	AST (≤40 U/L)	r-GT (6–42 U/L)	DB (≤8.0)	Ammonia (16–60)	WBC 5–20 × 10^9^/L	*N*% (45%–70%)	*L*% (30%–50%)	*M*% (4.0%–11.0%)	CRP (0–10 mg/L)	IL-6 <7 pg/ml	PCT (<0.5 ng/ml)	PT (11.5–14.0)	APTT (36–49)	Fib (1.7–4.1)	D-D (<0.5)	CnTI (<88 pg/ml)	Metabolic screen*
1	admission	7 h after fever	33	40	112	6.4		5.07	36.1	29.2	29.6	1.1								
	D5	5 days after fever	88	180	109	2.5		4.72	33.7	57	8.9	4.3								
	D10	10 days after fever	645	480	491	3.2		12.37	31.8	58.9	7.8	2.2							28.8	
	D11	11 days after fever					48								12.3	36.8	2.92	0.91		(−)
	D14	14 days after fever	125	44	283	2.8		11.45	23	66.9	7.9	0.2								
	D18/discharge																			
	8 days after discharge	follow up-1	32	73	163	2.1		9.64	28.4	54.1	10.3									
	15 days after discharge	follow up-2	38	81	119	2.2														
2	admission	30 h after fever	9	32	155	12.9		10.02	61.6	25.9	11.6	19.8							55.5	
	D2	3 days after fever						8.37	39.9	43.8	13.6	7.5								
	D6	7 days after fever	280	414	289	5		6.42	32.2	53.1	12	12.4		<0.1						
	D8	8 days after fever										1.87								
	D11	11 days after fever	197	242	596	3.6														
	D12/discharge																			
	13 days after discharge	follow up-1	10 (8–71)	26 (21–80)	325 (9–150)	2.1 (0–4)														
3	admission	2 days after fever	15	34	144	6.4		17.14	61.6	25.9	11.6	1.6								
	D2	4 days after fever	13	34	107	4.8		6.7	25.1	60.9	12.4	0.3								
	D6	8 days after fever	268	286	182	5.5		12.08	19.5	61.3	16.4	1.4								
	D10	12 days after fever	45	29	117	3.0						<0.5								
	D12/discharge																			
	9 days after discharge	follow up-1	11	21	54	4.5														
4	admission	1 days after fever	20	29	129	9.1		4.61	30.4	46.4	18.2	<0.5	6.02	0.13						
	D6	7 days after fever	151	234	151	<1.6		9.96	9.7	83.2	5.8	<0.5			10.4	37.4	2.03	2.36		
	D11	12 days after fever	97	40	212	4.5						<0.5								
	D12/discharge																			
	4 days after discharge	follow up-1	26	32	148	3.3		11.58	28.2	64.2	6.2									

### Case 2

A 7-day-old girl was admitted to our NICU because of a 30-hour intermittent fever with a peak temperature of 38.5°C. Her parents noted that she had a stuffy nose with clear rhinorrhea and choked when feeding over the past two days. She coughed occasionally but had sputum in her throat. Her appetite was unchanged without increasing work of breathing, vomiting, and diarrhea. Her caregivers had confirmed infection of SARS-CoV-2 several days ago. Her mother was healthy during pregnancy but detected fetal hydronephrosis with the right duplex kidney in the third trimester. The baby was born through an uneventful C-section delivery at a gestational age of 40w^+6^. The baby looked well after birth, and there was no evidence of early-onset sepsis. On physical examination, she had a temperature of 38.6°C and mild tachypnea of a respiratory rate of 50 breaths/min without retraction. Her lungs demonstrated coarse breath sounds without crackles and wheezes. The lab investigations showed that CRP was increased to 19.8 mg/L(0–10 mg/L) (see Table 1).

WBC, blood gas analysis, transaminase level, and bilirubin levels were all in the normal range. The blood culture for bacteria was negative. Chest x-rays suggested increased and blurred bilateral lung markings in both lung fields (see Figure 1). Cardiac ultrasound demonstrated a left-to-right shunt of 2.6 mm through a patent foramen ovale (PFO). Abdominal ultrasound showed mild bilateral hydronephrosis and calculus in the right kidney (3.1 mm × 2.7 mm), and there were no abnormalities in the liver, gallbladder, and spleen structures. PCR and quick antigen tests for SARS-CoV-2 from the throat swab were positive. There was no evidence of infection from other viruses [RSV, EBV, TORCH, ECHO virus, Coxsackie virus (CA16/CVB), influenza A/B, adenovirus, mycoplasma, chlamydia, HIV, Human metapneumovirus, rhinovirus, and hepatitis B/C virus]. Normal saline helped to clean the airway, and nasal drops were used to relieve nasal congestion. Moreover, superficial skin cooling was given when the baby was febrile, and piperacillin/tazobactam was administered for pneumonia.

The baby's body temperature declined to normal on the 2nd day after admission. The respiratory symptom alleviated over the following days. On the 6th day, the laboratory tests showed that the transaminase level increased significantly without abnormality of bilirubin and albumin (see Table 2 and Figure 2). Then, the hepatoprotective treatment of glycyrrhizin and bicyclol was administered.

ALT decreased by half on the 11th day. Given that the baby had recovered from fever and respiratory symptoms, she was discharged home with oral drugs of glycyrrhizin and bicyclol and was continued to be followed up in the outpatient department. Two weeks later, lab tests suggested that ALT and AST decreased to the normal range.

### Case 3

A 15-day-old girl was brought to the emergency center with a 2-day recurrent fever after contracting confirmed cases of COVID-19. The peak temperature was 38.1°C. Besides fever, she had a stuffy nose and a mild cough. She did not develop diarrhea and vomiting during the course. She had no complicated perinatal history. Her examination was febrile at 38.1°C with a respiratory rate of 48 breaths/min and oxygen saturation of 98%–100%. She had normal respiratory effort, and coarse breath sounds could be heard in all lung fields. Lab data of the WBC, CRP, blood gas, transaminase levels, and bilirubin levels were all in the normal range. The PCR test was positive for SARS-CoV-2 without other positive findings of other pathogens [RSV, EBV, TORCH, ECHO virus, Coxsackie virus (CA16/CVB), influenza A/B, adenovirus, mycoplasma, chlamydia, HIV, Human metapneumovirus, rhinovirus, and hepatitis B/C virus]. After admission, the baby was given the nebulization treatment of budesonide and ipratropium bromide, using normal saline and nasal drops to relieve nasal congestion and keep the nasal cavity clean (see Table 1).

The baby returned to normal temperature and developed frequent coughs over the days. Her lung examination demonstrated scattered crackles on the back side, and then, phlegm and wheezing sounds could be heard in the following days. Chest x-rays showed that bilateral lung markings increased and blurred, with patchy shadows on the right upper lung field (see Figure 1). The piperacillin/tazobactam for pneumonia was administered on the fourth day after admission. Since then, her symptoms and signs of respiratory improved gradually.

The baby got better on the 6th day after admission with wild nasal congestion. Laboratory tests showed that ALT and AST increased significantly (see Table 2 and Figure 2). She was also administered hepatoprotective treatment of glycyrrhizin and bicyclol. After a 2-week treatment, the liver function recovered totally.

### Case 4

A 24-day-old boy was admitted to the NICU for an 8-hour history of fever with a peak temperature of 38°C. The parents complained that the baby had a mild cough with sputum, and they noticed he seemed to have facial and lip cyanosis when feeding. The baby was lethargic and had a poor appetite, accompanied by watery diarrhea without emesis. He was born at a gestational age of 36w^+4^ via cesarean delivery. He had no remarkable perinatal history. His mother confirmed COVID-19 with fever and cough before he had symptoms. On physical examination, his temperature was 38°C with a respiratory rate of 46 breaths/min; blood pressure was in the normal range, and his SpO2 was 97% while feeding and breathing ambient air. Coarse breath without crackling sounds in all lung fields could be heard. No heart murmur was detected. Prominent reticulated mottling of the skin could be seen on the lower extremities, especially when he was febrile. Capillary refill time in the lower extremity was 2 s. The liver was palpated at 1 cm under the costal margin, and the spleen was not palpated. Laboratory data revealed that WBC, subsets proportion, CRP, procalcitonin (PCT), blood gas, electrolytes, transaminase, and bilirubin levels were all in the normal range. Blood culture was negative. No other apparent abnormalities were reported on the routine stool test. PCR test for the SARS-CoV-2 virus was positive. The common respiratory pathogens such as influenza A/B, RSV, parainfluenza, adenovirus, mycoplasma, and chlamydia were negative. Chest x-rays showed that bilateral lung markings increased and blurred (see Figure 1). The cardiac ultrasound was normal. The CRP tests were repeated, and sepsis was ruled out in the next few days. So, the baby was administered piperacillin/tazobactam for 36 h and nebulization treatment was given (see Table 1).

The fever lasted for 2 days, and the peak temperature was 38.6°C. The baby recovered with occasional cough and mild nasal congestion on the 5th day after admission. On the 6th day, the ALT and AST were significantly increased (see Table 2 and Figure 2). There was no evidence of infection for other pathogens related to liver injuries such as EBV, TORCH, ECHO virus, Coxsackie virus (CA16/CVB), and hepatitis B/C virus. He was also administered hepatoprotective treatment of glycyrrhizin and bicyclol.

The baby received hepatoprotective treatment for 5 days. On the 11th day after admission, repeated lab tests demonstrated that ALT and AST decreased to 97 U/L and 40 IU/L, respectively. Then he was discharged with hepatoprotective drugs. 4 days after discharge, the liver function went back to the normal range.

## Discussion

We presented four COVID-19 cases with hepatic injury for newborn infants in the SARS-CoV-2 variants Omicron epidemic. All patients had a normal liver function at the initial stage of the course. It was noted that hepatic dysfunction might have occurred 5 to 8 days after the first onset of fever, mainly characterized by moderate to severe transaminase elevation and without abnormalities in bilirubin level, blood ammonia, protein synthesis, lipid metabolism, and coagulation. All the patients received hepatoprotective therapy, and transaminase levels gradually decreased to the normal range after 1 to 2 weeks.

Liver involvement in adult patients with COVID-19 has been reported previously (12–14). As for Children suffering from COVID-19, they usually present mild or asymptomatic diseases. The liver involvement, characterized by the elevation of transaminases without hepatic synthetic dysfunction, could be seen in critically ill patients with multisystem inflammatory syndromes (MIS) secondary to SARS-CoV-2 infection (15, 16). Moreover, several cases in the literature reported that some children with an asymptomatic or mild presentation of COVID-19 disease developed long COVID-19 liver manifestations, including acute liver failure or acute hepatitis with cholestasis. The outcome varies widely from life-threatening and a need for liver transplant to total recovery after steroid treatment (17–20). Reports in the literature on liver involvement in neonates with SARS-CoV-2 infection were sparse. Stolfi et al. reported that a newborn patient infected with SARS-CoV-2 vertically without respiratory manifestations had an elevation of serum liver enzymes after birth (the peak of ALT and AST were 155 and 143 U/L, respectively) and gradually recovered on the day of life DOL10 (9). Another case from Sisman et al. presented a preterm infant with SARS-CoV-2 infection via intrauterine transmission, who developed a fever and mild respiratory disease on the second day of life, only had a slightly increased AST (64 U/L, normal range 10–35) and normal level of ALT (10). Compared with these two patients, babies in our case series were infected by contact with caregivers, the onset was late, and the liver injury was much more severe. Therefore, this is the first case series about moderate (ALT or AST is within 3 to 10 folds normal upper limit) to severe (ALT or AST >500 U/L or >10-fold normal upper limit) hepatitis in COVID-19 neonatal patients via horizontal transmission.

The potential mechanism of SARS-CoV-2 virus infection-associated liver injury could be attributed to hepatic tropism and direct cytopathic effects (21, 22). Hepatic biopsy pathology from a COVID-19 patient revealed that the SARS-CoV-2 virus could be seen in vessel lumens, endothelial cells of the portal vein, and the cytoplasm of hepatocytes, which leads to hepatocytic apoptosis (23, 24). Cytokine storm and MIS secondary to SARS-CoV-2 infection could also result in immune-mediated hepatocellular damage, albumin synthesis suppression, and cholestasis (21, 25, 26). Furthermore, hypoxia from acute respiratory and cardiac failure could also contribute to liver injury in critically ill patients (12, 13). Patients in this group are expected to be accompanied by hypoxic-ischemic myocardial injury. In addition, various drugs in clinical practice also contribute to liver injuries such as antiviral drugs, long-term antibiotics, corticosteroids, and antipyretic drugs (acetaminophen) (13, 22).

It was interesting that one patient in our group, suffering from longer fever duration and more severe liver injury, was co-infected with the SARS-CoV-2 virus and parainfluenza virus (PIV). To the best of our knowledge, PIV mainly causes respiratory tract illnesses such as bronchiolitis, pneumonia, and croup, and n on-respiratory manifestations are rare without reports of related liver involvement (27–30). Meanwhile, we ruled out the infection of other common viruses which could induce liver injuries such as RSV, EBV, TORCH, ECHO virus, Coxsackie virus (CA16/CVB), influenza A/B/H1N1/H3N2, adenovirus, mycoplasma, chlamydia, HIV, Human metapneumovirus, rhinovirus, and hepatitis A/B/C virus. Similar reports about severe acute hepatitis (SAH) of unknown etiology across multiple countries from January 2022 to June 2022 demonstrated that 91 of the 126 children (72%) suffered from the adenovirus infection; however, adenovirus alone is rarely associated with acute hepatic failure in healthy children (31). Further, Akash et al. reported that six of the eight children who developed liver failure and received liver transplants were all infected with the novel coronavirus (SARS-CoV-2 antibody positive); it was worth noting that adenovirus was detected in their whole blood samples but was undetected in the liver biopsies (32). Thus, it may speculate that virus co-infection may contribute to the severity of COVID-19 disease and aggravate related liver injury.

In addition, we also try to exclude other causes leading to liver injury. Firstly, all patients with mild or moderate fever were not administered antipyretic drugs for fever and other liver-injured medicines, so we could exclude the possibility of antipyretic drug-induced liver injury, which may be the confounding factor of liver involvement in some critically ill cases. The patient in Case 1 didn't receive antibiotics treatment, but patients in the other three cases were administered piperacillin/tazobactam in the short course. It was reported that piperacillin/tazobactam could lead to hepatotoxicity and drug-induced liver injury (DILI), which was the most common causative antibiotic of DILI in adults (33). However, the use of piperacillin/tazobactam in neonatal and pediatric patients is safe and effective as an empiric treatment for serious infections. Severe adverse events related to piperacillin/tazobactam use are hemolytic anemia, pustulous skin eruptions, drug hypersensitivity syndrome, and neutropenia and are time and dose-dependent (34, 35). Further study is needed to explore the incidence and severity of piperacillin/tazobactam-induced liver injury in a large population of neonatal and pediatric patients in a real-world setting. Secondly, the parents denied the related perinatal history and the mother's preview medical history of autoimmune disease. The babies did not present the abnormal direct bilirubin level, and all recovered without corticosteroid treatment in follow-up, so there was little evidence to support the diagnosis of autoimmune liver dysfunction. Thirdly, all the patients grew well without abnormal results of blood PH/base excess, lactate, blood ammonia, and pyruvic acid. Thus, metabolic liver disease could be ruled out. Lastly, none of the patients presented symptoms of MIS or hypoxia from acute respiratory distress, and two of them had normal levels of IL-6 and CnTI. Therefore, it can be concluded that the leading cause of acute hepatitis in these patients was the Omicron SARS-CoV-2 variant infection.

In our case series, we presented four COVID-19 neonate patients with liver involvement, describing the trend of transaminase over time and the short outcome. Based on these limited cases, it is hard to deeply explore and analyze population characteristics and risk factors for liver injury. We also lack reliable biomarkers to detect liver injury early in neonates with SARS-CoV-2 infection. Additionally, long-term follow-up is needed to ensure full recovery for the patients. Moreover, all the patients received hepatoprotective treatment, including glycyrrhizin, glutathione, or bicyclol. Glycyrrhizin (Magnesium isoglycyrrhizinate) is a hepatocyte protectant with anti-inflammatory effects and protection of the liver cell membrane. Glutathione is a strong antioxidant that could improve membrane stability to protect the liver cell membrane, promote detoxification, and repair enzyme activity. Bicyclol could protect the liver cell membrane by clearing free radicals, protect liver cell nuclear DNA from damage, and reduce the occurrence of apoptosis. These drugs, which are also used in the treatment of chronic viral hepatitis in adults, may help decrease the ALT and recover liver function.

This study is not a strict RCT trial or a well-designed cohort study. There are potential biases and cofounders which may affect the conclusion. In this case-serial report, no control group could balance some related factors, which is also our limitation in this study.

## Conclusions

Newborns are a high-risk group for COVID-19 in the condition of postnatal infection during the Omicron variants epidemic. Besides fever and respiratory symptoms, the clinical doctor should pay much attention to evaluating the risk of liver function injury after SARS-CoV-2 variants infection, which is usually asymptomatic and has a delayed onset. If the patient was co-infected with other pathogens, the symptoms and signs might be severe and long-lasting. It takes time for liver function recovery, so the patient should be followed up closely after discharge. Further research is needed to provide more evidence in the future.

## Data Availability

All relevant data is contained within the article: the original contributions presented in the study are included in the article/supplementary material, further inquiries can be directed to the corresponding author/s.
